# The impact of COVID-19 on illegitimate tasks across occupations in Sweden: a longitudinal observational study

**DOI:** 10.1186/s12889-026-27810-6

**Published:** 2026-05-20

**Authors:** Constanze Leineweber, Johanna Stengård, Robin S. Högnäs, Hanne Berthelsen

**Affiliations:** 1 Department of Psychology, Stockholm University, Stockholm, SE-106 91 Sweden; 2 Department of Psychology, Uppsala University, Uppsala, Sweden; 3 Centre for Work Life and Evaluation Studies (CTA), Faculty of Odontology, Malmö University, Malmö, Sweden

**Keywords:** COVID-19 pandemic, Illegitimate tasks, Psychosocial work environment, SLOSH

## Abstract

**Background:**

The COVID-19 pandemic led to major societal changes. Although the pandemic has significantly impacted the way we work, few studies have examined its effects on the prevalence of illegitimate tasks, i.e., tasks that are perceived as either unnecessary or unreasonable. Occupational groups were likely affected differently. This study investigates the prevalence of illegitimate tasks for registered nurses, teachers, IT specialists, and construction workers in pre-pandemic (2018), early-pandemic (2020), late-pandemic (2022), and post-pandemic (2024) periods.

**Methods:**

Data were collected from biennial waves of the Swedish Longitudinal Occupational Survey of Health (SLOSH), corresponding to pandemic periods (pre-, early-, late-, and post-pandemic). We included four different occupational groups that vary in terms of socially critical activities, the possibility of teleworking, and the level of social interactions required in the job: registered nurses (*n* = 385), teachers (*n* = 412), IT specialists (*n* = 353) and construction workers (*n* = 313). We employed Generalized Estimating Equations (GEE) to assess changes in illegitimate tasks over time.

**Results:**

Our longitudinal GEE models revealed that teachers reported a higher prevalence of illegitimate tasks than registered nurses, IT specialists, and construction workers. From the pre-pandemic period (2018) to the late-pandemic period (2022), all four occupational groups experienced a significant decrease in unreasonable tasks (adjusted for age), while unnecessary tasks decreased for all groups except teachers, for whom there was no significant change. By the post-pandemic period (2024), unnecessary tasks returned to pre-pandemic levels across all occupations, whereas unreasonable tasks remained at the lower, late-pandemic, level.

**Conclusions:**

The level of illegitimate work varies across different occupations, with a higher prevalence in female-dominated, human service fields compared to male-dominated technical roles. Addressing the growing care needs with more human resources will be challenging. One possible solution is to increase available working time by reducing illegitimate and especially unnecessary tasks. Overall, this study contributes to a better understanding of how an important aspect of the work environment, illegitimate tasks, shifted during a period of major societal change.

**Supplementary Information:**

The online version contains supplementary material available at 10.1186/s12889-026-27810-6.

## Background

The COVID-19 pandemic is marked as one of the most significant societal changes in recent times. As a result of the devastating effects of the virus spreading rapidly, intensive infection control measures were taken, jobs were reorganized, and new workplace tasks and routines were introduced, while working from home became much more common [1]. The work environments of healthcare workers and teachers were particularly affected by the pandemic. Healthcare workers faced an increased workload, which required rapid adjustments to focus more on core tasks. Meanwhile, teachers had to adapt to new teaching conditions and thus, changes to their pedagogical strategies. Some labor market sectors were able to continue more or less as usual. For example, findings from Sweden show that COVID-19 had only mild impacts on construction production with few consequences [2]. The IT sector also experienced few changes during the COVID-19 pandemic, as pre-pandemic telework was already common [3].

Many studies have examined the effects of the pandemic on the labor market, yet few empirical studies have examined its effects on work environment factors. One potential set of stressors influenced by labor market changes during the pandemic are “illegitimate tasks”— tasks that employees perceive as unreasonable or outside of the norms of what can reasonably be expected of them [4, 5]. Illegitimate tasks include both unnecessary and unreasonable tasks, and their prevalence varies between different occupational groups [6, 7]. Unreasonable tasks are those that an employee believes are someone else’s responsibility. Unnecessary tasks are tasks that are considered completely unnecessary, or those that require less effort if things were organized differently or other people made fewer mistakes. It is likely that the potential effect of the pandemic on illegitimate tasks differed across occupational groups.

The current study focuses on four occupational groups: registered nurses (hereafter referred to as nurses), teachers, IT specialists, and construction and manufacturing workers (hereafter referred to as construction workers). These occupations differ in several ways. Both nurses and teachers work in female-dominated, people-orientated jobs that require a lot of social contact. However, while teachers can carry out their work remotely (that is, the work can be fully or partly carried out on an alternative worksite other than the default place of work), this is almost impossible for nurses. Construction workers and IT specialists work in male-dominated occupations, and thus differ significantly from nurses and teachers. For example, the incidence of illegitimate work is lower among male-dominated technical occupations [7]. However, while construction workers have a job that cannot be done remotely, working remotely was already common among IT specialists before the pandemic.

### Illegitimate tasks – theoretical background

Research suggests that illegitimate tasks can lead to work-related stress and ill health [8]. The *Stress-as-Offense-to-Self (SOS) theory* [4, 5] posits that social threats to one’s self-esteem, particularly in the workplace, trigger stress. This stress emerges from situations that are perceived to offend a person’s self-image or identity, which is something that people generally strive to protect, thereby seeking to maintain a positive self-image. According to the SOS theory, illegitimate tasks send a social message of disrespect (i.e., stress as disrespect), and through this, imply a threat to the employee’s professional identity, which can lead to stress and ill health [5, 9]. Illegitimate tasks also take time and focus away from core tasks [10]. This may lead to stress from feelings of inadequacy, where one feels unable to fulfil the work requirements according to one’s own expectations and the norms of the profession (i.e., stress through insufficiency). Role theories and organizational justice have also been used to conceptualize illegitimate tasks [8]. However, empirical studies show that illegitimate tasks can explain unique variance in well-being even when considering similar factors such as role conflict, fairness in the division of labor, and social stress, suggesting that illegitimate tasks are a concept in their own right [5].

### Illegitimate tasks – previous research

Several studies have shown that illegitimate tasks are associated with declines in mental health [11, 12]. In turn, this has negative consequences for intrinsic motivation and one’s willingness to remain in the workplace [13–17]. In other words, illegitimate work not only affects individuals’ health, but it also has negative effects within the workplace. In the long term, it could become more difficult for organizations to recruit skilled people for important positions in the health and education sectors. Therefore, a change in level of illegitimate tasks could have significant societal implications.

Previous research has primarily examined micro (i.e., individual) and meso-level (i.e., work-related) factors as key determinants of illegitimate tasks [8]. For example, Aronsson and Mellner [6] found that gender, age, position and occupation were important factors associated with illegitimate tasks. Others have found differences in the prevalence of illegitimate tasks among managers in relation to a wide range of contextual work environment factors [18] and a positive association between work group size and the prevalence of illegitimate tasks among both managers and employees [19]. However, it is worth noting that such contextual work factors are not well-understood in the broader social context in which they arise [18] nor in the context of societal priorities and the organization of the public employment sector [20, 21]. Few studies have investigated the potential explanatory factors at the macro level, with the exception of a study in the Swedish healthcare sector, which examined the prevalence of illegitimate tasks by performance-based remuneration schemes among physicians [21]. In this study, physicians reported that a performance-based reimbursement system led to more illegitimate tasks. Overall, prior findings suggest that there may be important macro-level factors that also contribute to the prevalence of illegitimate tasks.

### COVID-19 and its implications for illegitimate tasks in various occupations

The COVID-19 pandemic, with its unplanned nature and its major impact on social relations and on the world of work, provides a unique naturalistic context for a longitudinal observational study of changes that would have been difficult to examine under ordinary circumstances. It affected the conditions under which work was organized and performed, and may have had different implications for different types of jobs. The pandemic forced the widespread adoption of social distancing and teleworking - defined here as ICT-enabled remote work - which changed the way people connect and collaborate. These “shocks” disrupted traditional norms and created valuable opportunities to study shifts in the work environment, including whether their effects on different occupations were temporary or signaled a lasting evolution in societal norms. Thus, the period before, during and after the pandemic offers a unique opportunity to better understand how the prevalence of illegitimate labor may have been affected by larger societal shifts.

Several studies have examined the impact of the COVID-19 pandemic on the labor market. Broadly, studies can be divided into three focus areas: (1) levels of occupational exposure to contagion; (2) potential for business continuity throughout containment measures, thanks in part to the possibility of working from home; (3) and finally, studies that examine the heterogeneous impact of the COVID-19 pandemic on the labor market [22]. Studies have shown, for example, that public sector employees in Finland who worked from home during the COVID-19 pandemic experienced increased control over working hours, procedural justice, and social capital in the workplace [23]. A report from the Swedish Work Environment Authority [1] showed that psychological workload increased during the pandemic, and that cooperation with, and support from, supervisors and colleagues was also affected. One in five employees reported increased social support during the pandemic, while slightly fewer felt that it had decreased. A relatively large proportion, just over a third, felt that cohesion and morale at work had deteriorated compared to before the pandemic. However, less is known about the impact of the pandemic on illegitimate tasks.

According to Flisi and Santangelo [22], three main occupational characteristics can explain differences in how the European labor market was affected by the pandemic, and the impact of the pandemic on different types of jobs: (i) whether a job was located in a socially critical sector, (ii) technical possibilities to work remotely in the given occupation and (iii) the level of social interaction required by a job.

The definitions of critical occupations vary between countries and over different periods. According to the Swedish Civil Defence and Resilience Agency [24] an essential activity is one, that maintains or ensures services, infrastructure, or activities necessary for societal functions, basic needs, values, or security. Flisi and Santangelo use recommendations published by the European Commission to define occupations as critical or non-critical [25]. According to this recommendation, socially critical occupations include, among others, healthcare occupations, teaching occupations, and information and communication technology occupations. Other occupations, such as construction work, are not considered socially critical. As for the other two criteria, these are based on whether or not a job can technically be performed remotely and, finally, on the amount of social interactions required in the performance of the work [22]. While many jobs can theoretically be performed remotely, some of them require a high degree of social interaction, which, when performed remotely, can degrade the quality of the work performed [22].

Based on Flisis and Santangelo’s classification, we selected four different occupations that differ in socially critical relevance, opportunities to work remotely, and the presence and importance of social interactions. Nurses, teachers and IT specialists are all defined as socially critical occupations. However, these three occupations differ regarding teleworking opportunities and the importance of social interactions. The health and education sectors require a high level of social interaction to maintain the quality of work. Still, while remote teaching is considered possible, teleworking is generally not considered feasible in health professions. IT specialists can often perform their jobs remotely, and fewer social interactions are required compared to professions in the health and education sectors. Finally, we focus on construction workers, whose work is generally not feasible to perform remotely and is to a high degree performed outdoors. Also, their work is not considered socially critical nor does it require many social interactions [22].

Table 1 shows the four selected occupations categorized based on Flisis and Santangelo’s proposed classification, taking into account that the possibility of teleworking was influenced by Sweden’s milder and more liberal pandemic policies compared to other EU countries [26]. The Swedish government recommended working from home if possible, self-imposed social distancing, self-monitoring for symptoms, staying home when ill, socializing outdoors and only with a small number of people, practicing good hand hygiene, and distance education at high school (in some periods) and universities, but these were recommendations not enforced measures.

**Table 1:** Categorization of nurses, teachers, IT specialists, construction workers. (adapted from Flisis & Santangelo’s categorization of the impact of the pandemic on the labor market.)

	Critical occupation	Technical telework ability (during the pandemic in Sweden)	Social interaction
Nurses	Yes	No (very limited opportunities)	High
Teachers	Yes	Yes (partly implemented in Sweden)	High
Construction workers	No	No	Low
IT-specialists	Yes	Yes (good opportunities)	Middle

Below, we provide a detailed description of the impact of the COVID-19 pandemic on these occupations. Starting with the healthcare job sector, which serves a critical societal function, nurses had minimal possibilities to shift to teleworking, as in-person social interaction with patients is required in most cases. Therefore, based on the three criteria above, it can be assumed that the way the job was performed did not change during the pandemic. However, media reports suggest that healthcare professionals felt that more time was spent on core tasks than in the pre-pandemic period [27]. However, the original source is no longer accessible for verification. This is in line with results from a Swedish study indicating a shift to a more patient-centered focus with fewer administrative tasks, more collaborative working relationships, more appreciation and meaningfulness in the work [28]. Intensive care nurses also found it very meaningful to be placed in a situation in which a strong focus on core tasks was required and competencies were crucial [29]. Overall, this points to an increased focus on the core mission in the healthcare sector, to provide compassionate care, during the pandemic. Therefore, we expect that the prevalence of illegitimate tasks decreased during the COVID-19 pandemic, but may have subsequently returned to pre-pandemic levels.

In terms of the education sector in Sweden, from the start of the pandemic, there was an explicit strategy to keep schools open as much as possible (Pashakhanlou, 2022). The argument was that parents in socially critical occupations should be able to perform their work and that students’ educational and social needs also should not suffer as a result of the pandemic [30]. However, secondary and postsecondary schools were closed early in the pandemic and distance learning was introduced [31]. Then, due to recommendations from the Public Health Agency in Sweden, some distance learning was also introduced in primary and lower secondary schools to meet the needs of teachers and students [30]. In a study conducted by the Swedish National Agency for Education, teachers reported a high workload during the pandemic, with sharp shifts between teaching on-site and teaching at a distance [31]. Major efforts were required to mitigate any potentially negative consequences for students.

Bergdahl and Nouri [32] show that structural issues emerged during the early process of shifting to distance learning during the COVID-19 pandemic in Sweden. Teachers and school leaders worked diligently to select digital technologies, while also considering GDPR requirements, evaluating students’ access to digital tools, and aligning choices with school policies. Bergdahl and Nouri further underscore that while preparedness to ensure continuity of education was initially slow, schools and teachers worked with tremendous effort to overcome the challenges. In addition, the Swedish National Agency for Education survey shows that most primary school teachers reported a higher workload during the pandemic than before the pandemic [31]. Additional work was required due to necessary online teaching and to get assignments to absent students. Teachers were given new tasks, including airing rooms, disinfecting desks and teaching materials, and ensuring that students kept their distance and washed and sanitized their hands [33]. We expect that this development increased the extent of illegitimate tasks among teachers during the pandemic, but would decrease to normal levels during the post-pandemic period.

The construction industry, on the other hand, was not similarly affected by major changes due to the COVID-19 pandemic, which may impact the number of illegitimate tasks [34]. Layoffs and cancellations were a bigger problem than changes in the work itself [35]. Thus, for construction workers, we do not anticipate substantial changes in the prevalence of illegitimate tasks due to the pandemic. Similarly, the IT sector can involve minimal face-to-face social interactions. A report from the European Commission suggests that, prior to the pandemic, telework was already common in the IT and communications sectors of the labor market [3]. Accordingly, we anticipate no or only minor changes in the prevalence of illegitimate tasks among IT specialists.

This leads us to the aim of the current study, which is to investigate the prevalence of illegitimate tasks during the pre-pandemic, early-pandemic, late-pandemic, and post-pandemic periods among nurses, teachers, IT specialists, and construction workers.

## Methods

### Sample

To examine changes in illegitimate tasks over time, we employed data from The Swedish Longitudinal Occupational Survey of Health (SLOSH), which is a panel study that aims to understand the relationships between labor market participation, work environment, retirement, and health (www.slosh.se). SLOSH was initiated in 2006 by the Stress Research Institute at Stockholm University, and data collection has since occurred every two years (since 2022, annually). The study population currently consists of participants in Statistics Sweden’s (SCB) work environment surveys (AMU) 2003–2021.

The present study includes SLOSH waves 2018 (pre-pandemic), 2020 (early-pandemic), 2022 (late-pandemic), and 2024 (post-pandemic), for which the response rates varied between 43 and 48%. In each wave, participants received one of two questionnaire versions depending on employment status (in work / not in work). The current study was restricted to individuals who responded to the questionnaire for those in paid work in at least two of the four waves (*n* = 9,151). Of these, participants were eligible if they reported working (not self-employed) as a nurse, teacher, IT specialist or construction/manufacturing worker in at least two waves and responds to questions about illegitimate tasks (see below) on these occasions. Applying these criteria resulted in a final analytic sample of 1,463 individuals: 797 people working in human service occupations (nurses, *n* = 385, and primary and secondary school teachers, *n* = 412) and 666 people working in technical occupations (construction workers, *n* = 313 and IT specialists, *n* = 353).

To examine the potential impact of non-response bias, we conducted an attrition analysis comparing participants who completed at least two waves (i.e., our study population) with those lost to follow-up after baseline on key characteristics. The groups did not differ significantly on age, sex, and baseline measures of unnecessary and unreasonable tasks, suggesting that selective attrition is unlikely to substantially bias the associations examined in this study.

### Measures

Illegitimate tasks were measured using the two dimensions of unreasonable and unnecessary tasks, each with four questions on a five-point scale from 1: *never* to 5: very often [5, 7]. An example question for unreasonable tasks is: “Do you have tasks to take care of which you believe should be done by someone else?” An example question for unnecessary tasks is: “Do you have tasks to take care of, which keep you wondering if they have to be done at all?” Mean values of all four questions for unreasonable and unnecessary tasks, respectively, were calculated for each individual and time point.

Sex and age were obtained from register data (LISA, The longitudinal integrated database for health insurance and labor market studies). Occupational information was collected in the SLOSH surveys using standardized questions, and then, for each wave, classified according to the Swedish Standardized Occupational Classification (SSYK 2012). In line with the aim of our study, we selected four different occupational groups that vary in terms of socially critical activities, the possibility of teleworking and the level of social interactions required in the job: nurses (SSYK 2220–2239), teachers (SSYK 2320, 2330 and 2341), IT specialists (SSYK 2510–2519) and occupations in construction and manufacturing (SSYK 7100–7233, again, referred to as construction workers).

### Analyses

To compare mean levels of unnecessary and unreasonable tasks between occupational groups at each wave, linear regression models were estimated with age and age squared as covariates. Pairwise comparisons between groups were conducted with Bonferroni correction. Data were analyzed (Stata version 18.0) using Generalized Estimating Equations (GEE), a method for longitudinal data that allows simultaneous analyses of variables measured at different time points [36]. GEE adjusts for the dependence of within-person observations over time by assuming a specific correlation structure between the outcome variable at different time points. Analyses were conducted using an unstructured working correlation structure, which is the least restrictive in which all correlations are allowed to vary. In addition, the parameters were estimated using the Huber-White sandwich estimator, which is robust to a misspecification of the correlation structure—it adjusts the standard errors to reflect the true variation in the estimates [37]. GEE analyses, stratified by occupational groups, included age (modelled with both linear and quadratic terms) as a covariate and time as the main predictor, and were estimated separately for unreasonable and unnecessary tasks. Given that the selected occupations are highly gender-segregated by design, gender was not included as a covariate in the models, as it is largely captured by occupational group membership. In total, the analytic sample included *n* = 1,226 individuals and *n* = 2,956 observations. This approach allowed us to examine changes over both shorter (2018–2020, 2020–2022, 2022-2024) and longer (2018–2024) time intervals within each occupational group.

## Results

Table 2 shows descriptive statistics by occupational group for the 2018 respondents. Women dominated human service occupations, while significantly more men worked in IT or construction. Furthermore, IT specialists were, on average, around three years younger than those in other occupations.

**Table 2:** Demographic background by occupation for 2018 (*n* = 1066)

	All	Nurses (*n* = 310)	Teachers (*n* = 303)	IT specialists (*n* = 211)	Construction workers (*n* = 242)
Gender (*n*, %)					
Men	464 (43.5)	26 (8.4)	65 (21.5)	143 (67.8)	230 (95.0)
Women	602 (56.5)	284 (91.6)	238 (78.6)	68 (32.2)	12 (5.0)
Age (mean, st.dev)	50.7 (8.6)	51.0 (8.9)	51.1 (8.4)	47.9 (8.2)	52.3 (8.5)

Table 3 shows the descriptive statistics (i.e., means and standard deviations) for unreasonable and unnecessary tasks by occupational group in the 2018 wave. Descriptive statistics for waves 2020–2024 are provided in the supplementary material (Additional file 1: Tables S1–S3). Cronbach’s alphas of the scales and mean differences between occupational groups are also reported in Table 3.

**Table 3:** Illegitimate tasks by occupation for 2018 (*n* = 1066)

	All	Nurses (*n* = 310)	Teachers (*n* = 303)	IT specialists (*n* = 211)	Construction workers (*n* = 242)	F(*p*-value)	Bonferroni
Unreasonable tasks (1–5)	2.53 (0.70)	2.59 (0.66)	2.75 (0.74)	2.26 (0.59)	2.39 (0.69)	22.36 (< 0.001)	T > *N* > C > IT*
Unnecessary tasks (1–5)	2.55 (0.74)	2.52 (0.75)	2.66 (0.77)	2.44 (0.71)	2.55 (0.70)	5.31 (< 0.001)	T > IT*
Alpha Unreasonable tasks	0.810	0.773	0.806	0.768	0.852	-	-
Alpha Unnecessary tasks	0.776	0.779	0.800	0.773	0.767	-	-

*p < .05. Values are unadjusted means (SD). Mean differences between occupational groups were tested using linear regression controlling for age (linear and quadratic terms), with Bonferroni-adjusted pairwise comparison. Values in parentheses represent standard deviations (SD)

### Change over time

**Fig. 1:**
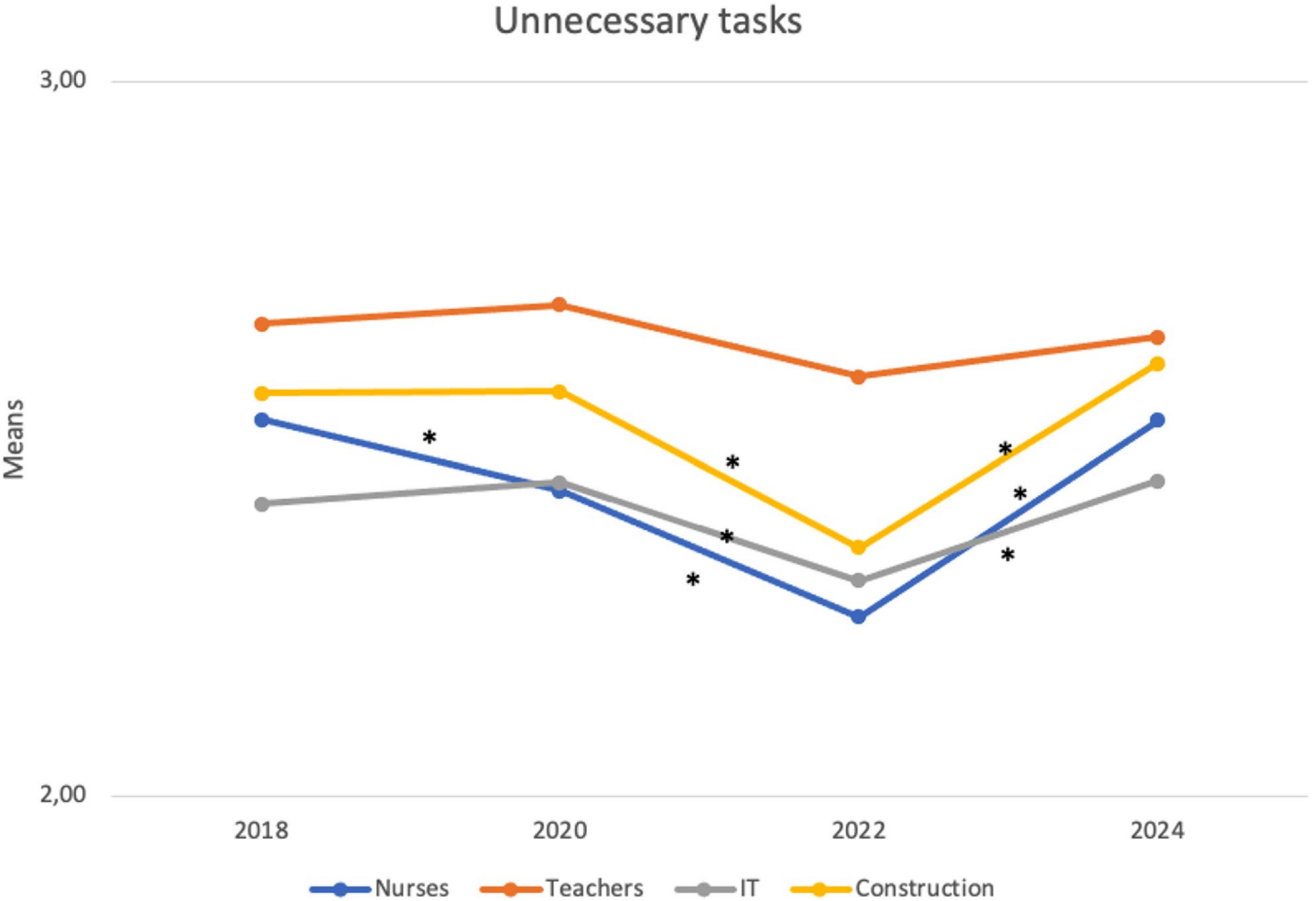
Adjusted mean scale scores for unnecessary tasks over time for each occupational group. Note: scale ranges from 1-5; * statistically significant (*p* <.05) change over time (adjusted for age)

**Fig. 2:**
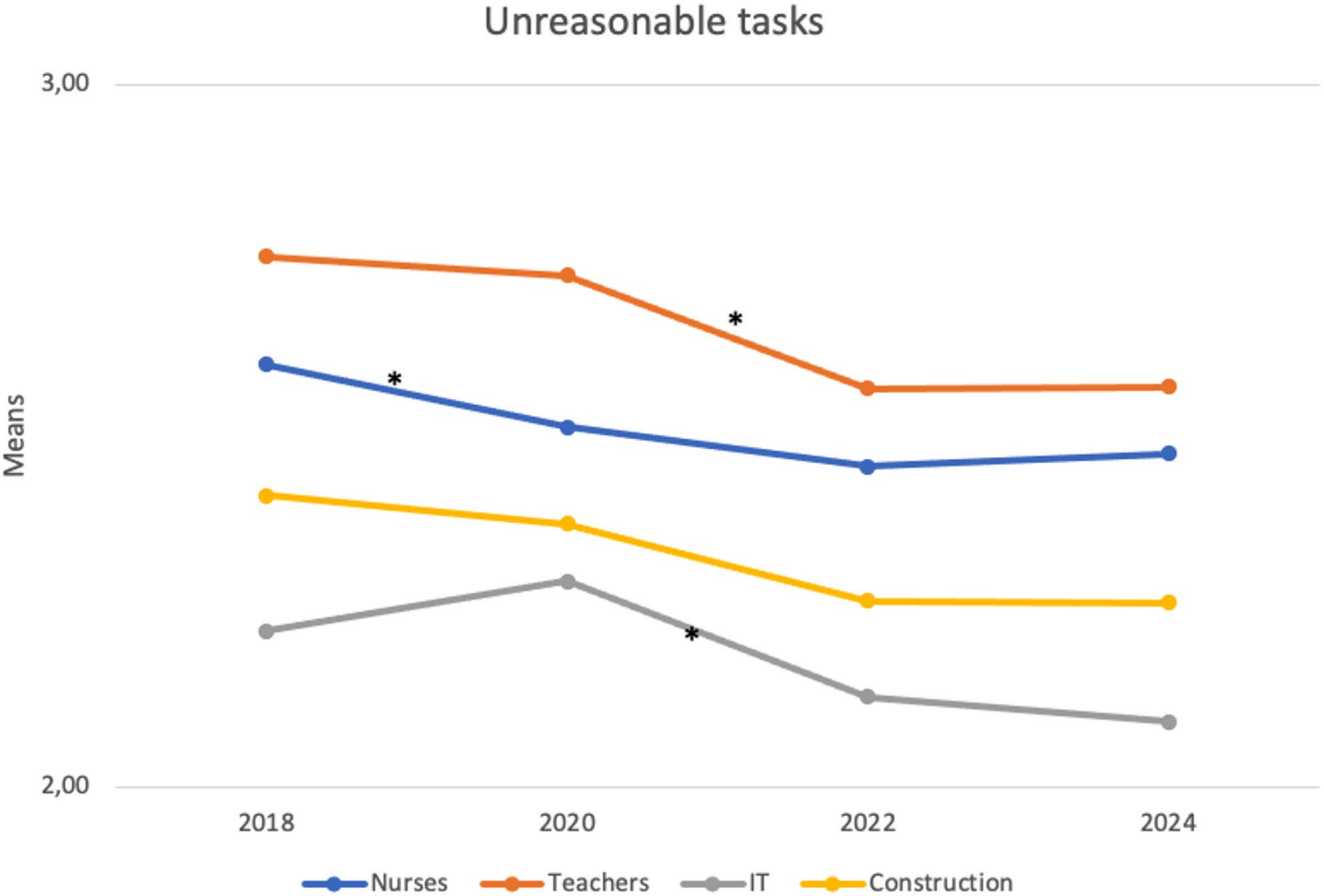
Adjusted mean scale scores for unreasonable tasks over time are shown in the graph for each occupational group. Note: scale ranges from 1-5; * statistically significant (p <.05) change over time (adjusted for age)

Table 4 presents the unstandardized regression coefficients for changes in unnecessary and unreasonable tasks over time. As shown, the level of unnecessary tasks differed significantly in none of the occupations between the pre-pandemic (2018) and post-pandemic (2024) periods, although some notable fluctuations were observed at the intermediate time points. For unreasonable tasks, although the levels were different across occupational groups, the patterns were similar, with decreases from the pre-pandemic period (2018) to the post-pandemic period (2024). Figures 1 and 2 illustrate how illegitimate tasks have changed over time by occupational category. Overall, although the occupational groups differed in their absolute levels—teachers reporting the highest levels of both unnecessary and unreasonable tasks, and nurses the second-highest levels of unreasonable tasks—the temporal pattern was similar across occupations.

**Table 4:** Unstandardized regression coefficients *b* (95% Confidence intervals) for change in unreasonable tasks and unnecessary tasks over time across occupations

	Unnecessary tasks b (95% CI)				Unreasonable tasks b (95% CI)			
	Nurses	Teachers	IT-specialists	Construction workers	Nurses	Teachers	IT-specialists	Construction workers
Pre- to Late-pandemic (Time 2018 → 2022)	− 0.23*** (-0.34; − 0.13)	− 0.03 (-0.12; 0.06)	− 0.10* (-0.19; − 0.00)	− 0.19*** (-0.31; − 0.07)	− 0.10* (-0.19; − 0.01)	− 0.15*** (-0.24; − 0.06)	− 0.12** (-0.21; − 0.04)	− 0.12* (-0.23; − 0.01)
Pre- to Early-pandemic (Time 2018 → 2020)	− 0.10* (-0.19; − 0.01)	0.04 (-0.04; 0.13)	0.03 (-0.06; 0.13)	0.01 (-0.10; 0.11)	− 0.08* (-0.15; − 0.01)	− 0.03 (-0.10; 0.05)	0.03 (-0.05; 0.11)	− 0.01 (-0.11; 0.08)
Early- to Late-pandemic (Time 2020 → 2022)	− 0.13** (-0.23; − 0.04)	− 0.07 (-0.16; 0.01)	− 0.13** (-0.22; − 0.04)	− 0.20*** (-0.32; − 0.08)	− 0.02 (-0.10; 0.06)	− 0.12** (-0.21; − 0.04)	− 0.16*** (-0.24; − 0.07)	− 0.11 (-0.22; 0.01)
Late- to Post-pandemic (Time 2022→ 2024)	0.24*** (0.14-0.34)	0.04 (-0.05; 0.13)	0.17*** (0.08; 0.26)	0.22*** (0.09; 0.35)	− 0.03 (-0.12; 0.07)	− 0.02 (-0.11; 0.07)	− 0.02 (-0.10; 0.06)	− 0.03 (-0.14; 0.09)
Pre- to Post-pandemic (Time 2018→ 2024)	0.01 (-0.10; 0.11)	0.01 (-0.10; 0.12)	0.07 (-0.04; 0.19)	0.03 (-0.10; 0.16)	− 0.13** (-0.22; − 0.03)	− 0.17*** (-0.27; − 0.07)	− 0.14** (-0.24; − 0.05)	− 0.15* (-0.27; − 0.03)

*p < .05; ** *p*<.01; *** *p*≤.001

Starting with unnecessary tasks, from the pre-pandemic period (2018) to the late-pandemic period (2022), reported levels of unnecessary tasks significantly decreased across all occupations (*b* = − 0.23, *p* ≤ .001 for nurses; *b* = − 0.10, *p* < .05 for IT specialists; and *b* = − 0.19, *p* ≤ .001 for construction workers), except for teachers (*b* = − 0.03, *p* = .516). These results indicate that the major decrease in unnecessary tasks occurred between the early-pandemic period (2020) and the late-pandemic period (2022). However, levels of unnecessary tasks subsequently rebounded to their pre-pandemic levels by the post-pandemic period (2024) (*b* = 0.24, *p* ≤ .001 for nurses; *b* = 0.17, *p* ≤ .001 for IT specialists; and *b* = 0.22, *p* ≤ .001 for construction workers).

Turning now to unreasonable tasks, from the pre-pandemic period (2018) to the late-pandemic period (2022), reported levels of unreasonable tasks significantly decreased across all occupations (*b* = − 0.10, *p* < .05 for nurses; *b* = − 0.15, *p* ≤ .001 for teachers; *b* = − 0.12, *p* < .01 for IT specialists; and *b* = − 0.12, *p* < .05 for construction workers). Between the late-pandemic period (2022) and the post-pandemic period (2024), levels remained stable.

Taken together, observed changes across both unnecessary and unreasonable tasks were modest in magnitude. Significant coefficients ranged from –0.10 to –0.24 on a 1–5 scale (standardized effect sizes: d = 0.13–0.32), accounting for 5–7% of occupational baseline means. While these effects are small by conventional standards, their consistent direction across occupational groups is noteworthy.

## Discussion

In this study, we investigated how the prevalence of illegitimate tasks evolved from the pre-pandemic to the post-pandemic periods among four distinct occupational groups: registered nurses, teachers, IT specialists, and construction workers. Apart from differences in the development of unnecessary and unreasonable tasks, our findings suggest apparent differences in the pre-pandemic prevalences of unnecessary and unreasonable tasks between occupational groups, especially for unreasonable tasks.

Starting with our primary aim, to examine change in illegitimate tasks over time, we did not find any change in unnecessary tasks between the pre-pandemic period (2018) and the post-pandemic period (2024) regardless of the occupation. We did, however, observe a sharp decrease in unnecessary tasks among nurses, IT specialists, and construction workers between the early-pandemic (2020) and late-pandemic (2022) periods, followed by an increase to pre-pandemic levels between 2022 and 2024. This partly mirrors results from Swedish work environment surveys [38], which showed a decrease in illegitimate tasks between 2015 and 2021 (i.e., measured there as ‘other things taking time from the main job’). Turning to unreasonable tasks, we found an overall decrease across all occupational groups from the pre-pandemic period, which stabilized between the late-pandemic period (2022) and the post-pandemic period (2024).

While the absolute magnitude of these changes was modest, several features highlight their practical relevance. Most notably, the near-complete reversal of the pandemic-era reduction in unnecessary tasks—especially among nurses—suggests that these shifts reflect genuine responses to changing work conditions, not random variation. The consistency of reductions in unreasonable tasks across all four occupational groups further supports this interpretation, as such convergent change is unlikely to occur by chance across diverse professional contexts. Additionally, it is unsurprising that meaningful shifts in established work characteristics, such as illegitimate tasks, appear as small-to-moderate effects, since these traits typically evolve gradually at the population level.

The diverging developments in unnecessary and unreasonable tasks are difficult to explain, and may be explained by a number of factors across occupational groups. The current findings suggest that, despite little change over time, teachers generally experience a higher frequency of both unnecessary and unreasonable tasks compared to the other occupational groups that we studied. Still, as noted, we observed a decrease in unreasonable tasks among teachers from the pre-pandemic period (2018). One potential explanation is a growing awareness of the challenges that teachers face and a desire for them to concentrate on their primary role, which is to teach. This is consistent with findings from the Swedish National Agency for Education [39] showing that the number of teaching assistants consistently increased between the 2017/18 and 2022/23 school years. However, the expansion of teaching assistants did not seem to impact the unnecessary tasks reported by teachers. Efforts to address and reduce these tasks are warranted, and this may require efforts beyond hiring more teachers or assistants given the consistently high prevalence of unnecessary tasks. An alternative strategy to address this problem could be policies that explicitly target unnecessary tasks in an effort to reduce them.

Among nurses, one possible explanation for the observed decrease in illegitimate tasks between the pre-pandemic (2018) and late-pandemic (2022) periods, may be that administrative tasks among healthcare professionals decreased de facto during the pandemic, as physicians previously expressed [40]. A report from the Swedish National Health Competence Council further suggests that more decisions in daily work during the pandemic were delegated to the profession [41]. The situation required flexibility, which resulted in, among other things, more administrative efficiency and an overall reduction in administrative burden. The results from the current study imply that a greater proportion of healthcare staff could focus on the more important and meaningful task of working closer to patients and many administrative tasks could be removed without major consequences. However, while unreasonable tasks decreased during the pandemic, we observed a rebound in unnecessary tasks for nurses (and across occupations) between the late-pandemic (2022) and post-pandemic (2024) periods. Overall, these results may point to clarified roles during the pandemic which led to fewer unreasonable tasks, but this did not appear to be the case for unnecessary tasks.

The pattern of change among IT specialists and construction workers was consistent with that of nurses, although the unreasonable task levels were lower. Among construction workers, these patterns are not easily explained. One possibility is that the pandemic’s travel restrictions and the resulting labor shortages forced employers to increase the productivity of existing staff by reducing the time and resources spent on less relevant tasks. Another possibility is that employers may have become more focused on productivity, thereby reducing tasks not directly linked to project progress. However, further research is needed to better understand the mechanisms underlying these changes for construction workers.

With respect to the IT sector, it may be that the decrease in unnecessary tasks during the pandemic was linked to the possibility of teleworking. Few or no requirements for in-person meetings can be beneficial as in-person meetings may be perceived as time away from more important tasks. Consistent with this argument, it has been suggested that teleworkers often choose to work away from the office simply to avoid interruptions, which are believed to improve productivity [42]. Another study on digital communication patterns in North America, Europe, and the Middle East further suggests that, although meetings have become more frequent, they have also become shorter. Consequently, employees spent less total time in meetings at the end of the COVID-19 pandemic [43]. Our results suggest that this may no longer be the case, and that we may have returned, at least in part, to a pre-pandemic meeting culture. On the other hand, online-meetings may be more common in the post-pandemic period. It may also be the case that some unnecessary tasks not performed during the pandemic were performed more frequently in the post-pandemic period after having been placed on hold. More research is needed to unpack these processes as well as whether teleworking has changed our meeting culture.

It is also important to address the occupational gradient; teachers consistently report the highest levels of illegitimate tasks, both unreasonable and unnecessary, over our observation period. Teachers and nurses reported significantly more unreasonable tasks than IT specialists and construction workers, but not more unnecessary tasks—nurses reported similar or fewer unnecessary tasks than other occupational groups in most years. These findings are thus consistent with Aronsson & Mellner’s [6], who found that illegitimate tasks were more common among school principals than vicars and private sector employees. Also, a report from Arena Idé [44] shows that social care, education, and public administration sectors stand out in terms of tasks that are considered unnecessary.

The skewed gender distribution in our occupational groups was intentional, reflecting our purposeful selection of professions across different domains. While prior research links gender to the experience of illegitimate tasks [6], we argue that observed changes over time are more plausibly driven by shifts in work demands, role boundaries, and organizational conditions—rather than by the gender composition of the workforce. Emphasizing gender as the primary explanation risks overlooking substantive transformations in the nature of professional work within these sectors.

The high prevalence of unreasonable tasks among teachers and nurses is worrying, not least because of the risk of sick leave, which has high rates in Sweden, but also because of high attrition rates and reduced interest in educational programs in these sectors. Indeed, illegitimate tasks can lead to significant threats to self-esteem [45], which may impact mental and physical health. Both teachers and nurses are key staff in welfare work that requires intensive social interaction, and these professionals are mainly employed in the public sector. Public sector employees generally feel, to a much greater extent than private sector employees, that their work is useful for society; and compared to many other professions, there is much pride attached to their work [46]. However, according to the SOS theory, the high prevalence of illegitimate tasks may signal a lack of appreciation for these employees [47]. This argument is supported by Furåker [46], who found that public sector employees perceive their conditions as worse than private sector employees in several respects, including earnings and job security.

In summary, our main findings suggest that the COVID-19 pandemic may indeed have impacted the frequency of illegitimate tasks, although the mechanisms underlying this decrease are unclear. On the one hand, the COVID-19 pandemic reduced unreasonable tasks. Perhaps people recognized that many tasks that they previously performed were not actually required. Also, telework may have helped to clarify occupational roles. On the other hand, our findings for unnecessary tasks suggest a return to old ways of working following the pandemic. The post-pandemic return to office may have reintroduced tasks perceived as unnecessary, such as commuting or redundant in-person meetings, potentially contributing to the observed increase. The lessons from the COVID-19 pandemic show that we need managers who reduce their need for control, promote trust-based leadership, and clearly communicate their expectations to employees [33]. Organizational measures that lead to greater decision-making space and influence, and clarify roles and tasks, have the potential to permanently decrease illegitimate tasks. Our results also underscore that attempts to reduce the burden of documentation demands and other tasks regarded as unnecessary are highly warranted.

### Strengths and limitations of the study

A strength of this work is that the data were initially drawn from the work environment surveys, and thus begins with a representative sample of the Swedish population. Another strength of the study is that our measures are repeated over time, which allows us to follow a large group of people over several years. To our knowledge, apart from a similar study that we published in Swedish [48], the current study is among the first panel studies on changes in the prevalence of illegitimate work across occupational groups. We have previously shown that BITS exhibits measurement invariance between these specific occupational groups [7], thus allowing group mean comparisons. In a previous Swedish study, BITS also has been shown to be invariant over time [49], which future studies should address in terms of the two underlying dimensions. Another strength of this study is that we examine two dimensions of illegitimate tasks: unnecessary and unreasonable tasks. We can conclude that although these two types of illegitimate tasks are highly correlated, their prevalence changes in different ways over time, showing the importance of considering these two dimensions separately in order to more efficiently highlight possible policy target areas.

This study, like all studies, also has limitations. First, it is important to note that the data collection was not specifically designed to measure the impact of the COVID-19 pandemic. The 2020 data collection coincided with the early-pandemic period, spring to summer of 2020, and it is not possible to know how much the situation at the time, nor whether pre-pandemic conditions in people’s lives, influenced survey responses. Another possible limitation is that there have been minor changes in the measurement of illegitimate tasks over time; for example, the location of the scale in the survey was slightly different from 2022, and the response categories slightly changed. However, these changes were minimal, and we have no reason to believe that this had any significant impact on our results. Lastly, non-response, attrition and aging, to some extent, may bias our results as many participants reached pension age during the study period. Thus, our sample may overrepresent the most satisfied and healthy among older workers, that is, there is a risk of a “healthy-worker” effect, since participants who dropped out are more likely to have experienced adversity, such as health problems, job loss, burnout, or career transitions. Requiring responses on at least two of the four waves likely limited this risk to some extent. Response rates in the included waves (43–48%) were lower than in earlier waves of SLOSH, reflecting a general decline in survey participation over time. Our attrition analyses indicated that participants included in the study were broadly comparable to those lost to follow-up on key baseline characteristics; nevertheless, selective non-response cannot be fully excluded. Methods such as inverse probability weighting or multilevel modeling could be applied in future studies to further address potential bias due to attrition.

### Implications for research and practice

Previous research has shown that performing illegitimate tasks can lead to poor health, decreased performance, and a desire to leave one’s profession or workplace [8]. These effects have high costs for both individuals and organizations. Therefore, it is beneficial for both individuals and organizations to reduce the number of illegitimate tasks. The current study observed particularly high levels of illegitimate tasks among teachers and nurses, professions that have current shortages and are at risk of future shortages. Despite an overall decrease in unreasonable tasks over time, the levels remained higher among teachers and nurses in the post-pandemic period. Studies indicate that the demand for trained teachers and nurses will significantly increase in the coming years, especially due to many professionals nearing retirement age. In Sweden, the estimated total recruitment needed for full-time teachers and preschool teachers by 2035 is approximately 131,000 [50]. The exact shortage of nurses is unclear, but all 21 regions of Sweden have reported a shortage of specialist nurses, and 17 of them reported a shortage of nurses [51]. Shortages in these professions threatens the Swedish welfare system. Although, these problems are not restricted to Sweden, as population aging and the rising number retirees in the coming years is indeed a problem that many Western countries currently face. A stable workforce of teachers and nurses is important in equal measure in all countries. To achieve this, employers must show appreciation through increasing trust, reducing administration, and consequently reducing the number of employee’s illegitimate tasks.

## Conclusions

This study shows that illegitimate tasks have generally decreased during the pandemic with a rebound of unnecessary tasks in the post-pandemic period. Thus, this work contributes to a better understanding of how an important aspect of the work environment was influenced during a time of major societal change. Our findings further underscore the importance of occupational variation over time, and that the prevalence of unreasonable tasks in female-dominated human service occupations is higher than in male-dominated technical and manual occupations. Among teachers in particular, both dimensions of these tasks are consistently higher than the other occupational groups over time, which needs more attention both in policy and research. Human service professions are central to the welfare of a society, and without them, the welfare state cannot be maintained. Meeting the increased care needs with increased human resources will be challenging—one possibility is to free up working time absorbed by illegitimate, and above all unnecessary, tasks.

## Supplementary Information

Supplementary Material 1.

## Data Availability

Given restrictions from the ethical review board and considering that sensitive personal data are handled, it is not possible to make the data freely available. Access to the data may be provided to other researchers in line with Swedish law and after consultation with the Stockholm University legal department. Requests for data, stored at the Stress Research Institute, Department of Psychology, should be sent to registrator@su.se with reference to ‘Illegitimate tasks 2023-15’ or directly to the corresponding author.

## Abbreviations

AMU: Arbetsmiljöundersökning
COVID-19: CoronaVirus Disease of 2019
GEE: Generalized Estimating Equations
IT: Information Technology
ITC: Information and Communication Technology
LISA: The longitudinal integrated database for health insurance and labor market studies
SCB: Statistiska Centralbyrån
SLOSH: Swedish Longitudinal Occupational Survey of Health
SOS: Stress-as-Offence-to-Self
SSYK: Standard för svensk yrkesklassificering
