# Supplementary material for: The impact of COVID-19 on illegitimate tasks across occupations in Sweden: a longitudinal observational study
Source: BMC Public Health. 2026 May 20;26:1569. doi: 10.1186/s12889-026-27810-6 (PMC13188769; doi:10.1186/s12889-026-27810-6)
Supplement: Supplementary file 1 — Supplementary Material 1. [file 12889_2026_27810_MOESM1_ESM.docx]

**Additional file 1**

**Table S1.** Illegitimate tasks by occupation for 2020 (n=1088)

|  | All | Nurses  (n=311) | Teachers  (n=311) | IT specialists (n=236) | Construction workers (n=230) | F  (p-value) | Bonferroni* |
| --- | --- | --- | --- | --- | --- | --- | --- |
| Unreasonable tasks (1-5) | 2.50 (.69) | 2.50 (.60) | 2.73 (.76) | 2.32 (.63) | 2.36 (.67) | 15.71 (<.001) | Teachers > all; Nurses > IT |
| Unnecessary tasks (1-5) | 2.53 (.75) | 2.42 (.70) | 2.69 (.79) | 2.46 (.69) | 2.56 (.77) | 6.30 (<.001) | Teachers > Nurses, IT |

NOTE: **p* < .05. Values are unadjusted means (SD). Mean differences between occupational groups were tested using linear regression controlling for age (linear and quadratic terms), with Bonferroni-adjusted pairwise comparison. Values in parentheses represent standard deviations (SD).

**Table S2.** Illegitimate tasks by occupation for 2022 (n=870)

|  | All | Nurses  (n=207) | Teachers  (n=247) | IT specialists (n= 260) | Construction workers (n=156) | F(p-value) | Bonferroni* |
| --- | --- | --- | --- | --- | --- | --- | --- |
| Unreasonable tasks (1-5) | 2.35 (.71) | 2.44 (.70) | 2.56 (.72) | 2.15 (.64) | 2.25 (.68) | 13.14 (<.001) | Teachers, Nurses > IT, Constr. |
| Unnecessary tasks (1-5) | 2.38 (.75) | 2.24 (.77) | 2.58 (.75) | 2.33 (.71) | 2.33 (.74) | 9.18 (<.001) | Teachers > all |

NOTE: **p* < .05. Values are unadjusted means (SD). Mean differences between occupational groups were tested using linear regression controlling for age (linear and quadratic terms), with Bonferroni-adjusted pairwise comparison. Values in parentheses represent standard deviations (SD).

**Table S3.** Illegitimate tasks by occupation for 2024 (n=882)

|  | All | Nurses  (n= 223) | Teachers  (n=240) | IT specialists (n=261) | Construction workers (n=158) | F(p-value) | Bonferroni* |
| --- | --- | --- | --- | --- | --- | --- | --- |
| Unreasonable tasks (1-5) | 2.35 (.74) | 2.46 (.68) | 2.57 (.77) | 2.12 (.70) | 2.24 (.73) | 19.45 (<.001) | Teachers, Nurses > IT, Constr. |
| Unnecessary tasks (1-5) | 2.55 (.76) | 2.51 (.73) | 2.64 (.81) | 2.47 (.73) | 2.58 (.76) | 6.23 (<.001) | Teachers > IT |

NOTE: **p* < .05. Values are unadjusted means (SD). Mean differences between occupational groups were tested using linear regression controlling for age (linear and quadratic terms), with Bonferroni-adjusted pairwise comparison. Values in parentheses represent standard deviations (SD).
